# Supplementary material for: Galanin receptor 1 expressing neurons in hippocampal-prefrontal circuitry modulate goal directed attention and impulse control
Source: Neuropsychopharmacology. 2026 Feb 11;51(8):1442–53. doi: 10.1038/s41386-026-02360-y (PMC13291240; doi:10.1038/s41386-026-02360-y)
Supplement: Supplementary file 1 — All supplementary materials Fig S1- S9 [file 41386_2026_2360_MOESM1_ESM.pdf]

Figure Legends – Supplementary material

Supplementary Figure 1. Expression of galanin fibers in the vPFC and the vHC.

(a) Schematic representing the location of the area analyzed in the PFC. **(b)** Microphotograph shows the distribution of galanin fibers and terminals in the PFC subdivisions (scale bar: 200 µm). **(c)** Magnified image of the galanin-containing fibers and terminals in the IL (scale bar: 200 µm). **(d)** Quantification of the mean density of galanin immunofluorescence in the three PFC subdivisions (Cg1: 0.43 ± 0.035, PrL: 0.43 ± 0.04, IL: 0.47 ± 0.04; au: arbitrary unit; N = 5 animals, 15 sections per region). Bar chart represents mean ± SEM. Dots represent individual animals. **(e)** Schematic representing the location of the area analyzed in the HC. **(f)** Microphotograph shows the distribution of galanin fibers and terminals in the vHC subdivisions (scale bar: 200 µm). **(g)** Magnified image of the galanin-containing fibers and terminals in the vCA1 (scale bar: 200 µm). **(h)** Quantification of the mean density of galanin immunofluorescence in the vHC subdivisions (CA1: 2.32 ± 0.22, CA2: 2;51 ± 0.10, CA3: 2.06 ± 0.11, DG: 2.40 ± 0.18; au: arbitrary unit; N = 1 animal, 4 to 15 sections per region). Bar chart represents mean ± SEM. Dots represent individual animals.

Supplementary Figure 2. Statistics for the main behavioral outcomes assessed in the 5-choice task for the optogenetic experiment.

Supplementary Figure 3. Statistics for the latency measures in the 5-choice task for the optogenetic experiment.

Supplementary Figure 4. Proportion of response types between laser conditions.

(a) Proportion of response types in non-stimulated and stimulated trials for vPFC-ChR2 animals (rats n = 8). **(b)** Proportion of response types in non-stimulated and stimulated trials for vPFC-tdTomato animals (rats n = 7). **(c)** Proportion of response types in non-stimulated and stimulated trials for vHC-ChR2 animals (rats n = 12). **(d)** Proportion of response types in non-stimulated and stimulated trials for vHC-tdTomato animals (rats n = 5).

Supplementary Figure 5. Comparison of MO and PrL/IL optical stimulation on behavioral responses.

(a) Schematic of brain section showing location of viral expression in the medial orbital region (MO) with corresponding photomicrograph. **(b)** Proportion of response types in non-stimulated and stimulated trials for animals with optic fiber in the MO brain region (rats n = 8). **(c-f)** Behavioral effects of optically stimulating GalR1-expressing cells in the MO region relative to PL/IL region on accuracy ($F(1, 14) = 0.362$, $p = 0.557$, $\eta^2_p = 0.025$), omissions ($F(1, 14) = 6.369$, $p = 0.025$, $\eta^2_p = 0.312$), premature responses ($F(1, 14) = 7.44$, $p = 0.016$, $\eta^2_p = 0.347$), and correct response latencies ($F(1, 14) = 0.842$, $p = 0.374$, $\eta^2_p = 0.057$); (MO: n = 8; IL: n= 8). Error bars represent SEM. * $p < 0.05$ (paired t-test between conditions after significant interaction effect in mixed ANOVA)

Supplementary Figure 6. Location of optical fibers in the vPFC and vHC for the optogenetics experiments.

Supplementary Figure 7. Location of optical fibers in the vPFC and vHC for the photometry experiments.

Supplementary Figure 8. Statistics for GCaMP signal in the 5-choice task for the photometry experiment.

Supplementary Figure 9. Statistics for Multinomial logistic regression to determine whether pre-stimulus calcium activity predicted behavioral outcome.

Figure S1

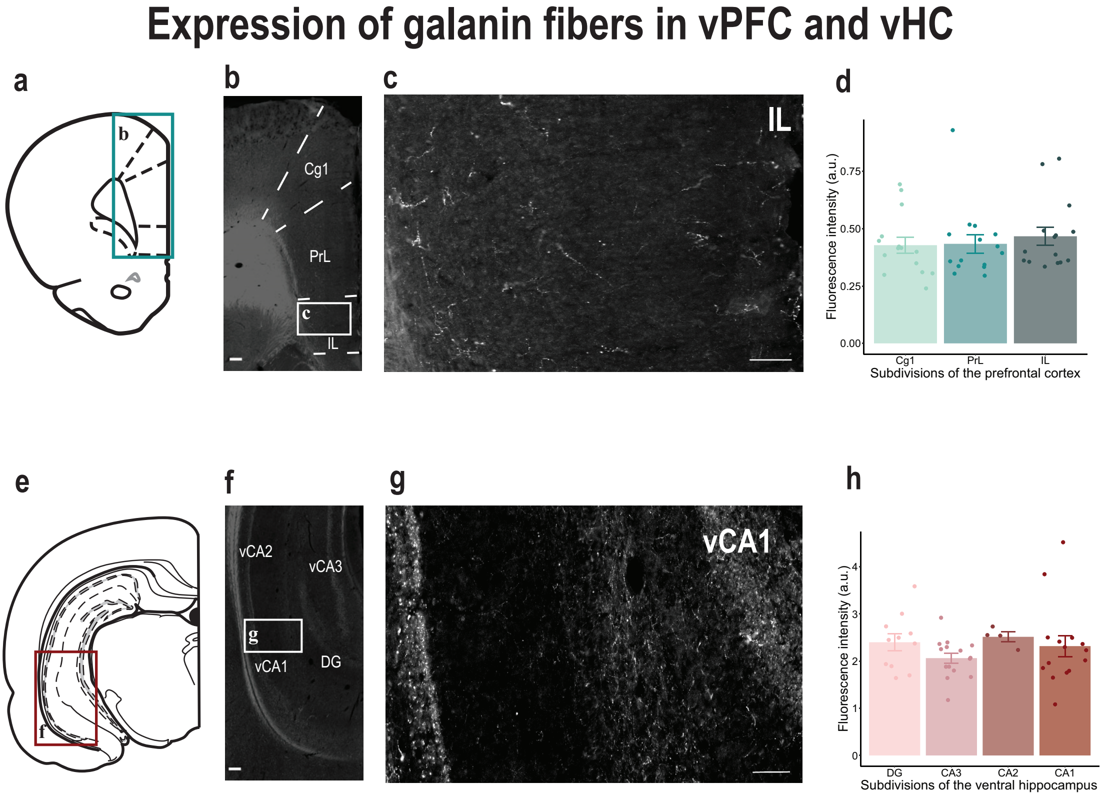
Expression of galanin fibers in vPFC and vHC
a
b
c
d
b
Cg1
PrL
c
IL
IL
Fluorescence intensity (a.u.)
0.00
0.25
0.50
0.75
Cg1
PrL
IL
Subdivisions of the prefrontal cortex
e
f
g
h
f
vCA2
vCA3
g
vCA1
DG
vCA1
Fluorescence intensity (a.u.)
0
1
2
3
4
DG
CA3
CA2
CA1
Subdivisions of the ventral hippocampus

Figure S2

Statistics of the parameters measured in the 5-choice task optogenetic experiments

		Accuracy	Correct response	Incorrect response	Omission	Premature response
**ChR2 in vPFC**						
Laser OFF	Mean	**82.02081**	**51.45122354**	**10.98895492**	**17.64606213**	**19.91375941**
	SEM	2.901515	5.53019645	2.003617865	5.105644181	5.989359496
Laser ON	Mean	**68.98836**	**35.98660714**	**15.28822761**	**43.48285623**	**5.242309013**
	SEM	4.986862	7.77719399	3.375468469	10.38658831	2.015927265
**tdTomato in vPFC**						
Laser OFF	Mean	**89.21182**	**63.4662332**	**7.358951268**	**15.35178713**	**13.8230284**
	SEM	2.637418	5.568235637	1.798810977	5.2688695	3.921215118
Laser ON	Mean	**91.90784**	**65.16947289**	**5.376452789**	**19.07976087**	**10.37431346**
	SEM	1.769553	5.277246771	1.187370978	4.452511768	3.314086832
Statistical comparison between groups (ChR2 and tdTomto) and conditions (Laser): (Mixed ANOVA, interaction)						
	F value	8.104	2.835	2.727	7.248	2.598
	p value	0.014	0.116	0.123	0.018	0.123
	η_p^2	0.384	0.179	0.173	0.358	0.167

		Accuracy	Correct response	Incorrect response	Omission	Premature response
ChR2 in MO						
Laser OFF	Mean	**89.1865**	**59.67896893**	**6.826730244**	**14.52529734**	**18.96900349**
	SEM	2.637418	5.307878602	1.421312652	4.927990041	4.10098683
Laser ON	Mean	**72.94635**	**45.88361308**	**12.02889976**	**17.25603009**	**24.83145707**
	SEM	7.475957	9.682159026	2.441670829	5.310827873	7.779375757
Statistical comparison between groups (PFC and MO) and conditions (Laser): (Mixed ANOVA, interaction)						
	F value	0.362	0.032	0.553	6.369	7.44
	p value	0.557	0.861	0.469	0.024	0.016
	η_p^2	0.025	0.002	0.038	0.313	0.347
Statistical comparison between groups (MO and tdTomato) and conditions (Laser): (Mixed ANOVA, interaction)						
	F value	10.592	3.855	8.878	0.078	3.509
	p value	0.006	0.71	0.011	0.784	0.084
	η_p^2	0.449	0.229	0.406	0.006	0.213

		Accuracy	Correct response	Incorrect response	Omission	Premature response
ChR2 in vHC						
Laser OFF	Mean	**85.5132**	**57.70791888**	**11.09966969**	**15.95211515**	**15.24029629**
	SEM	2.90706	3.392341087	1.876534431	2.452586439	3.240376983
Laser ON	Mean	**79.16721**	**51.86716637**	**12.37572558**	**25.74606523**	**10.01104282**
	SEM	3.49986	4.445038384	1.550673059	3.175536439	2.263969261
tdTomato in vHC						
Laser OFF	Mean	**90.70627**	**69.60419998**	**7.182922929**	**11.23568916**	**11.97718793**
	SEM	1.370147	2.739733298	1.190402456	3.272632679	0.927973488
Laser ON	Mean	**87.83151**	**67.25955986**	**9.328444508**	**7.462850442**	**15.94914519**
	SEM	2.05587	1.635296674	1.57792892	3.105930942	3.112960774
Statistical comparison between groups (ChR2 and tdTomto) and conditions (Laser): (Mixed ANOVA, interaction)						
	F value	0.023	0.303	0.04	6.672	1.939
	p value	0.882	0.59	0.844	0.021	0.184
	η_p^2	0.002	0.02	0.003	0.308	0.114

Figure S3

**Statistics of the latencies measured in the 5-choice task optogenetic experiments**					
		**Correct response latency**	**Incorrect response latency**	**Premature response latency**	**Magazine latency**
**ChR2 in vPFC**					
Laser OFF	Mean	**0.8365**	**1.942375**	**3.98875**	**1.189375**
	SEM	0.044755686	0.215749353	0.200171333	0.068890425
Laser ON	Mean	**1.15225**	**3.1695**	**3.6152**	**1.2925**
	SEM	0.141407561	0.415298946	0.228427696	0.082934785
**tdTomato in vPFC**					
Laser OFF	Mean	**0.848857143**	**1.747**	**4.165571429**	**1.118428571**
	SEM	0.07374132	0.539940164	0.161744486	0.055153836
Laser ON	Mean	**0.801857143**	**1.937666667**	**4.033833333**	**1.162142857**
	SEM	0.06668277	0.403783865	0.174455793	0.061122605
Statistical comparison between group and conditions (Mixed ANOVA, interaction)					
	F value	8.175	0.141	0.841	0.241
	p value	0.013	0.713	0.376	0.632
	η_p^2	0.386	0.011	0.061	0.018
		**Correct response latency**	**Incorrect response latency**	**Premature response latency**	**Magazine latency**
**ChR2 in vHC**					
Laser OFF	Mean	**0.893416667**	**2.203083333**	**3.998**	**1.314**
	SEM	0.038191991	0.235732432	0.153232286	0.095016187
Laser ON	Mean	**0.989**	**2.745333333**	**4.036727273**	**1.307416667**
	SEM	0.047291168	0.165140818	0.131714533	0.094689561
**tdTomato in vHC**					
Laser OFF	Mean	**0.7836**	**1.133287563**	**4.073**	**1.1836**
	SEM	0.024019992	0.506821606	0.095375049	0.036428835
Laser ON	Mean	**0.7504**	**1.5354**	**4.2398**	**1.1946**
	SEM	0.048171153	0.259181905	0.075960121	0.052013075
Statistical comparison between group and conditions (Mixed ANOVA, interaction)					
	F value	2.117	1.871	0.099	0.103
	p value	0.166	0.191	0.007	0.753
	η_p^2	0.124	0.111	0.099	0.007

Figure S4

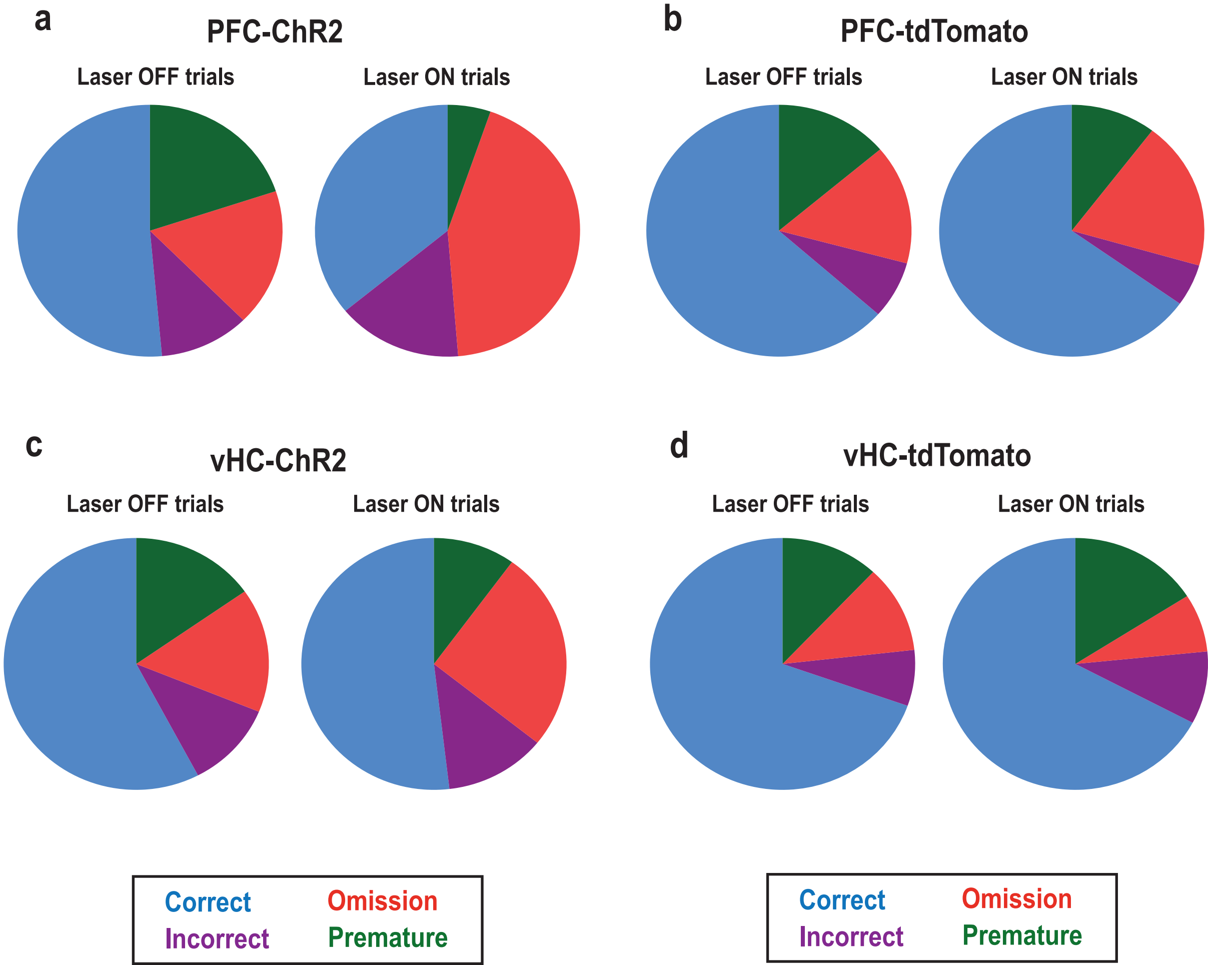

a
PFC-ChR2
Laser OFF trials
Laser ON trials
b
PFC-tdTomato
Laser OFF trials
Laser ON trials
c
vHC-ChR2
Laser OFF trials
Laser ON trials
d
vHC-tdTomato
Laser OFF trials
Laser ON trials
Correct
Omission
Incorrect
Premature
Correct
Omission
Incorrect
Premature

Figure S5

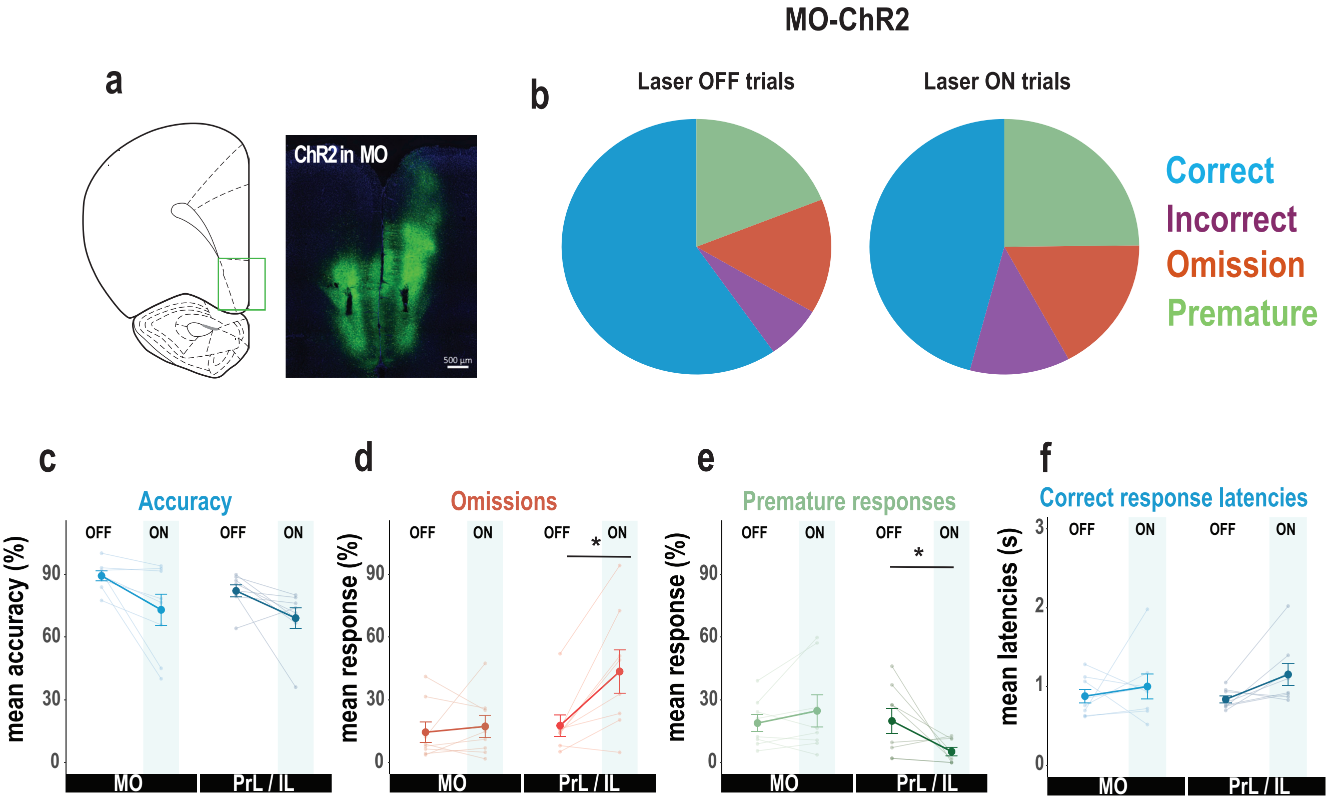
MO-ChR2
a
ChR2 in MO
500 µm
b
Laser OFF trials
Laser ON trials
Correct
Incorrect
Omission
Premature
c
Accuracy
mean accuracy (%)
OFF
ON
MO
PrL / IL
d
Omissions
mean response (%)
e
Premature responses
f
Correct response latencies
mean latencies (s)

Figure S6

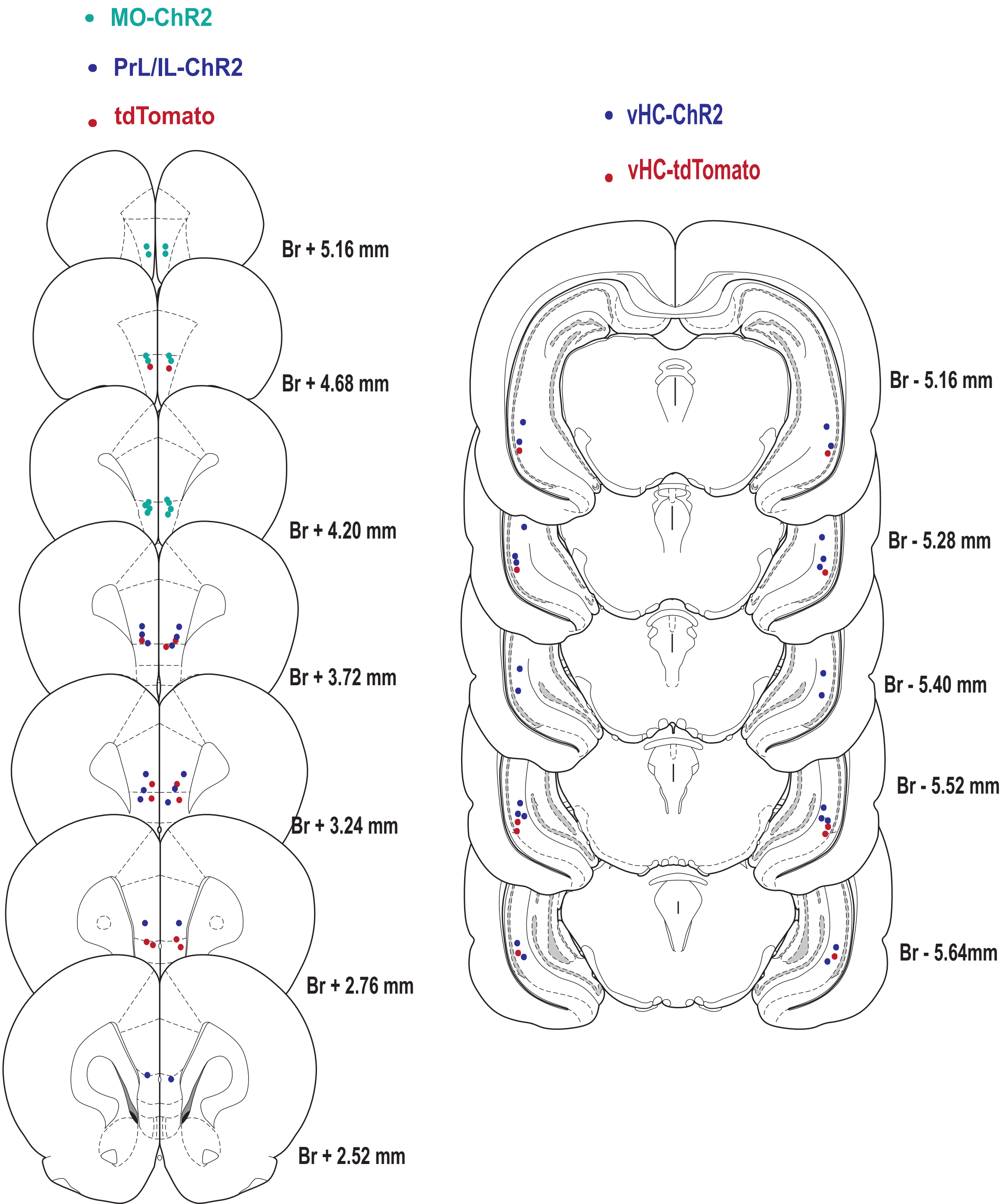

MO-ChR2
PrL/IL-ChR2
tdTomato
Br + 5.16 mm
Br + 4.68 mm
Br + 4.20 mm
Br + 3.72 mm
Br + 3.24 mm
Br + 2.76 mm
Br + 2.52 mm
vHC-ChR2
vHC-tdTomato
Br - 5.16 mm
Br - 5.28 mm
Br - 5.40 mm
Br - 5.52 mm
Br - 5.64mm

Figure S7

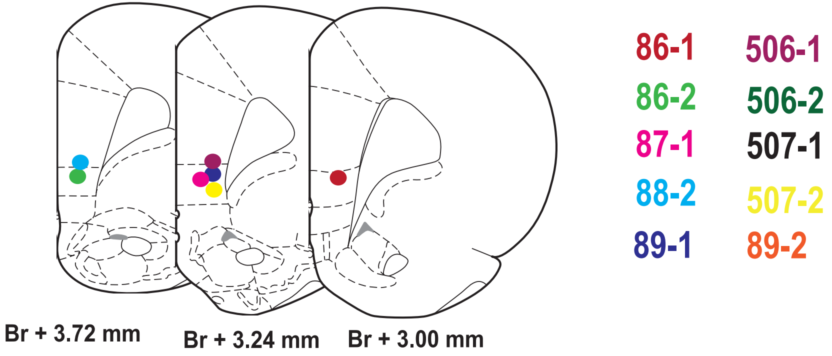
86-1
506-1
86-2
506-2
87-1
507-1
88-2
507-2
89-1
89-2
Br + 3.72 mm
Br + 3.24 mm
Br + 3.00 mm

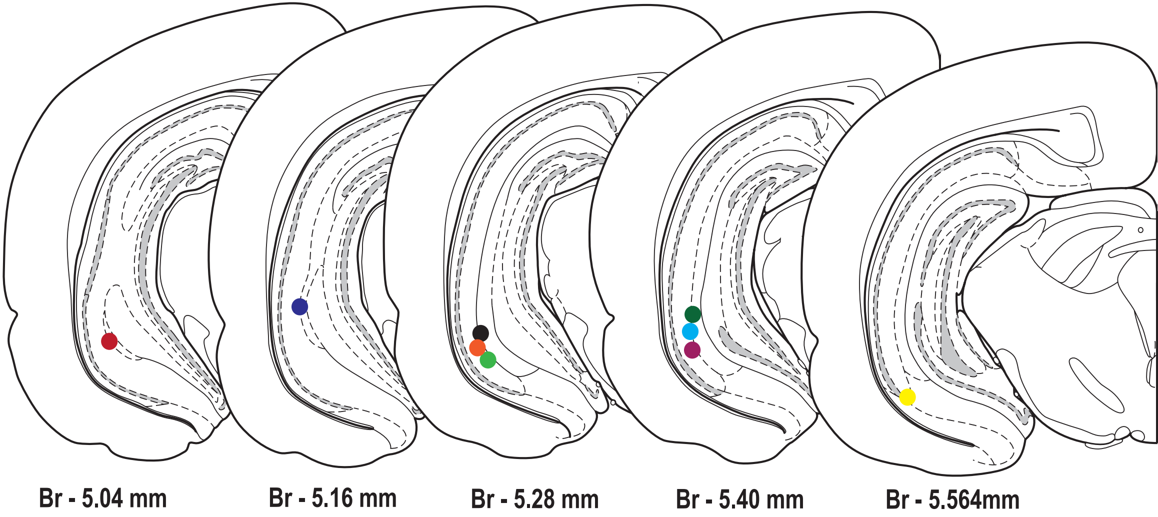
Br - 5.04 mm
Br - 5.16 mm
Br - 5.28 mm
Br - 5.40 mm
Br - 5.564mm

Figure S8

Statistics of the GCaMP signal in the 5-choice task photometry experiments

GCaMP in vPFC

**2s before trial initiation**	**Statistical comparison (One-way ANOVA) between responses**	
	F value	1.826
	p value	0.169
**1 s before trial initiation**	**Statistical comparison (One-way ANOVA) between responses**	
	F value	1.968
	p value	0.146
**Trial initiation**	**Statistical comparison (One-way ANOVA) between responses**	
	F value	4.328
	p value	**0.014**
	**Post-hoc comparison (p values)**	
	correct vs incorrect	0.355
	correct vs omission	0.082
	correct vs premature	**0.01**
	incorrect vs omission	0.835
	incorrect vs premature	0.305
	omission vs premature	0.782
**1s after trial initiation**	**Statistical comparison (One-way ANOVA) between responses**	
	F value	3.154
	p value	**0.043**
	**Post-hoc comparison (p values)**	
	correct vs incorrect	0.502
	correct vs omission	0.205
	correct vs premature	**0.03**
	incorrect vs omission	0.926
	incorrect vs premature	0.408
	omission vs premature	0.77
**2 s after trial initiation**	**Statistical comparison (One-way ANOVA) between responses**	
	F value	4.274
	p value	**0.015**
	**Post-hoc comparison (p values)**	
	correct vs incorrect	0.232
	correct vs omission	**0.02**
	correct vs premature	**0.029**
	incorrect vs omission	0.618
	incorrect vs premature	0.719
	omission vs premature	0.998
**3 s after trial initiation**	**Statistical comparison (One-way ANOVA) between responses**	
	F value	3.731
	p value	**0.025**
	**Post-hoc comparison (p values)**	
	correct vs incorrect	0.44
	correct vs omission	**0.044**
	correct vs premature	**0.036**
	incorrect vs omission	0.574
	incorrect vs premature	0.516
	omission vs premature	1
**4 s after trial initation**	**Statistical comparison (One-way ANOVA) between responses**	
	F value	6.649
	p value	**0.007**
	**Post-hoc comparison (p values)**	
	correct vs incorrect	0.148
	correct vs omission	**0.005**
	incorrect vs omission	0.148
**cue presentation**	**Statistical comparison (One-way ANOVA) between responses**	
	F value	5.538
	p value	**0.011**
	**Post-hoc comparison (p values)**	
	correct vs incorrect	0.116
	correct vs omission	**0.009**
	incorrect vs omission	0.427

GCaMP in vHC

**2s before trial initiation**	**Statistical comparison (One-way ANOVA) between responses**	
	F value	0.243
	p value	0.866
**1 s before trial initiation**	**Statistical comparison (One-way ANOVA) between responses**	
	F value	0.115
	p value	0.95
**Trial initiation**	**Statistical comparison (One-way ANOVA) between responses**	
	F value	0.326
	p value	0.807
**1s after trial initiation**	**Statistical comparison (One-way ANOVA) between responses**	
	F value	0.402
	p value	0.753
**2 s after trial initiation**	**Statistical comparison (One-way ANOVA) between responses**	
	F value	0.907
	p value	0.449
**3 s after trial initiation**	**Statistical comparison (One-way ANOVA) between responses**	
	F value	1.181
	p value	0.332
**4 s after trial initation**	**Statistical comparison (One-way ANOVA) between responses**	
	F value	1.202
	p value	0.318
**cue presentation**	**Statistical comparison (One-way ANOVA) between responses**	
	F value	1.35
	p value	0.278

Figure S9

Multinomial logistic regression tables for calcium acitivity during the pre-stimulus interval

a

Predictors	χ2	p value
vPFC AUC	19.985	0.001
vHC AUC	2.468	0.481

b

Region	Reference category	Categories	B	Std error	Wald χ2	p value	e^B	95% CI
**vPFC**	**Correct**	Incorrect	-0.128	0.073	3.083	0.079	0.88	0.763 __ 1.015
		Omission	-0.154	0.074	4.353	**0.037**	0.857	0.742 __ 0.991
		Premature	-0.159	0.074	4.62	**0.032**	0.853	0.738 __ 0.986
	**Incorrect**	Correct	0.128	0.073	3.083	0.079	1.136	0.985 __ 1.311
		Omission	-0.026	0.025	1.143	0.285	0.974	0.928 __ 1.022
		Premature	-0.031	0.025	1.594	0.207	0.969	0.924 __ 1.017
	**Omission**	Correct	0.154	0.074	4.353	**0.037**	1.167	1.009 __ 1.348
		Incorrect	0.026	0.025	1.143	0.285	1.027	0.978 __ 1.077
		Premature	-0.005	0.011	0.197	0.657	0.995	0.974 __ 1.017
	**Premature**	Correct	0.159	0.074	4.62	**0.032**	1.172	1.014 __ 1.355
		Incorrect	0.031	0.025	1.594	0.207	1.032	0.983 __ 1.083
		Omission	0.005	0.011	0.197	0.657	1.005	0.984 __ 1.026

c

Region	Reference category	Categories	B	Std error	Wald χ2	p value	e^B	95% CI
**vHC**	**Correct**	Incorrect	-0.005	0.01	0.204	0.652	0.995	0.976 __ 1.016
		Omission	0.011	0.011	0.956	0.328	1.011	0.989 __ 1.033
		Premature	-0.001	0.011	0.013	0.909	0.999	0.978 __ 1.02
	**Incorrect**	Correct	0.005	0.01	0.204	0.652	1.005	0.985 __ 1.025
		Omission	0.016	0.012	1.789	0.181	1.016	0.993 __ 1.039
		Premature	0.003	0.01	0.118	0.731	1.003	0.984 _ 1.023
	**Omission**	Correct	-0.011	0.011	0.956	0.328	0.989	0.968 __ 1.011
		Incorrect	-0.016	0.012	1.798	0.181	0.985	0.962 __ 1.007
		Premature	-0.012	0.011	1.142	0.285	0.988	0.966 _ 1.01
	**Premature**	Correct	0.001	0.011	0.013	0.909	1.001	0.98 __ 1.022
		Incorrect	-0.003	0.01	0.118	0.731	0.997	0.977 __ 1.016
		Omission	0.012	0.011	1.142	0.285	1.012	0.99 _ 1.035
